# Fat mass and obesity-related (FTO) shuttles between the nucleus and cytoplasm

**DOI:** 10.1042/BSR20140111

**Published:** 2014-10-22

**Authors:** Pawan Gulati, Edward Avezov, Marcella Ma, Robin Antrobus, Paul Lehner, Stephen O’Rahilly, Giles S. H. Yeo

**Affiliations:** *MRC Metabolic Diseases Unit, University of Cambridge Metabolic Research Labs, Wellcome Trust-MRC Institute of Metabolic Science, Addenbrooke's Hospital, Cambridge CB2 0QQ, U.K.; †NIHR Cambridge Biomedical Research Centre, Addenbrooke's Hospital, Cambridge, U.K.; ‡Department of Medicine, Cambridge Institute for Medical Research, University of Cambridge, Cambridge, U.K.

**Keywords:** Exportin 2, fat mass and obesity-associated protein (FTO), nucelocytoplasmic shuttling, obesity, AA, amino acid, co-IP, co-immunoprecipitation, FLIP, fluorescence loss in photobleaching, GFP, green fluorescent protein, HEK-293 cells, human embryonic kidney cells, IP, immunoprecipitation, MEF, mouse embryonic fibroblast, mTOR, mammalian target of rapamycin, SNP, single nucleotide polymorphism, WT, wild-type, XPO2, Exportin 2

## Abstract

SNPs (single nucleotide polymorphisms) on a chromosome 16 locus encompassing *FTO*, as well as *IRX3*, *5*, *6*, *FTM* and *FTL* are robustly associated with human obesity. FTO catalyses the Fe(II)- and 2OG-dependent demethylation of RNA and is an AA (amino acid) sensor that couples AA levels to mTORC1 (mammalian target of rapamycin complex 1) signalling, thereby playing a key role in regulating growth and translation. However, the cellular compartment in which FTO primarily resides to perform its biochemical role is unclear. Here, we undertake live cell imaging of GFP (green fluorescent protein)-FTO, and demonstrate that FTO resides in both the nucleus and cytoplasm. We show using ‘FLIP’ (fluorescence loss in photobleaching) that a mobile FTO fraction shuttles between both compartments. We performed a proteomic study and identified XPO2 (Exportin 2), one of a family of proteins that mediates the shuttling of proteins between the nucleus and the cytoplasm, as a binding partner of FTO. Finally, using deletion studies, we show that the N-terminus of FTO is required for its ability to shuttle between the nucleus and cytoplasm. In conclusion, FTO is present in both the nucleus and cytoplasm, with a mobile fraction that shuttles between both cellular compartments, possibly by interaction with XPO2.

## Abstract 2

Exportin interacts with FTO and this interaction might be involved in the nucelocytoplasmic shuttling of FTO in the cell.

## INTRODUCTION

GWAS (Genome-wide association studies) have indicated that SNPs (single nucleotide polymorphisms) on a chromosome 16 locus encompassing *FTO*, as well as *IRX3*, *5*, *6*, *FTM* and *FTL* are robustly associated with human obesity, with carriers of the risk alleles reported to have increased appetite [1–3]. Data from mouse models demonstrate that at least three of these genes play a role in the regulation of body size and composition. Targeted deletion of *Irx3* in mice results in reduced body size and resistance to HFD-induced obesity [4], whereas mice heterozygous for a deletion in *Ftm* (also *Rpgrip1l*) results in increase of body-weight [5]. Transgenic manipulation of *Fto* in mice supports the notion that it also regulates body size, with overexpression resulting in obesity [6], whereas *Fto* null mice [7] and humans homozygous for a loss-of-function allele [8] display post-natal growth retardation and have high early mortality.

*In vitro*, recombinant FTO is able to catalyse the Fe(II)- and 2OG-dependent demethylation of single-stranded nucleic-acids, with a preference for RNA [9–11]. We have previously reported that FTO is an AA (amino acid) sensor that couples AA levels to mTORC1 (mammalian target of rapamycin complex 1) signalling, thereby playing a key role in regulating growth and translation [12]. Cells lacking FTO display decreased activation of the mTORC1 pathway, decreased rates of mRNA translation and increased autophagy all of which are likely to contribute to the phenotype of stunted growth seen in humans and mice homozygous for loss-of-function mutations in FTO [12].

The question remains however, over the cellular compartment in which FTO primarily resides to perform its biochemical role. Initial immunofluorescense studies by us and others appeared to show that FTO was localized to the nucleus with no signal detected in the cytoplasm [9,13]. Subsequently, we then showed using cellular fractionation, that in addition to the nucleus, we were able to detect a substantial proportion of FTO residing in the cytoplasmic fraction [12]. These two apparently conflicting results can be explained by the fact that fixing cells with organic solvents could disrupt the dynamic physiological distribution of FTO in the cell [14]. To this end, the purpose of this study is to establish the cellular localization of FTO in live cells and how this localization might be regulated. We show, using live cell imaging coupled with ‘FLIP’ (fluorescence loss in photo bleaching) that FTO resides in both the nucleus and cytoplasm and that a mobile FTO fraction shuttles between the two compartments. We identify XPO2 (Exportin 2) as a binding partner of FTO, which might be involved in the shuttling of FTO between nucleus and cytoplasm.

## EXPERIMENTAL

*Reagents:* Anti-rabbit FTO antibody, raised against recombinant full-length FTO, was kindly provided by Professor Roger Cox, Mammalian Genetics Unit, MRC Harwell, Oxford, U.K.; mouse monoclonal anti-FTO antibody was from PhosphoSolutions, Colorado, U.S.A.; rabbit anti-actin was from Abcam; rabbit anti-Flag, rabbit anti-GFP (green fluorescent protein) and anti-Flag M2 magnetic beads were from Sigma; Histone and tubulin antibodies were from Cell Signaling; rabbit anti-XPO2 antibody was from Assay Biotech. Protein Sepharose-A and -G and HRP (horseradish peroxidase)-conjugated secondary antibodies were from Amersham. All other reagents not mentioned above were from Sigma.

*Cell culture and transfections:* COS7, MEFs (mouse embryonic fibroblasts) and HEK-293 cells (human embryonic kidney cells) were maintained in DMEM (Dulbecco's modified minimal essential medium) supplemented with 10% (v/v) FCS. For ectopic expression studies, transfections were performed using CalPhos kit (for HEK-293 cells) from ClonTech and Neon System (for COS7) from Invitrogen according to the manufacturer's protocol.

### Fluorescence imaging of live COS7 cells

Zeiss 510 Meta laser scanning confocal system with Plan-Apochromat ×63 oil immersion lens (NA=1.6) coupled to a microscope incubator that maintained standard tissue culture conditions (Okolab), was used for live cell imaging. A series of 40–60 images with 2 s intervals was acquired (excitation at 488 nm, emission 505–530 nm). Bleaching of a selected ROI (region of interest) in image 3 was performed using 100% laser power (488 nm, 300–600 iterations). Mean intensity was measured inside/outside the apparent nucleus.

*IP (immunoprecipitation) and glycerol gradient fractionation:* FTO and Flag IPs as well as sub-cellular fractionations were performed as previously reported [12]. The truncated C-terminal FTO was generated by PCR from FTO full-length cDNA (primer sequences provided on request) and sub-cloned into pFLAG-CMV-2 vector (SIGMA) at EcoRV/SalI sites. Similarly, the truncated N-terminal FTO was generated by PCR and sub-cloned into pFLAG-CMV-2 vector at HindIII/SalI sites. All constructs were confirmed by direct nucleotide sequencing.

*MS analysis:* Excised gel bands from SDS–PAGE were subjected to in-gel tryptic digestion and the resulting eluted peptides were analysed by LC-MSMS using an OrbiTrap XL coupled to a nanoAcquity. Raw MS data were processed using MaxQuant v.1.0.13.13 Quant module and the resulting .msm files searched against SwissProt v.57.1 using Mascot Daemon 2.3. Carbamidomethyl (C) was defined as a fixed modification with deamidation (NQ), oxidation (M) and acetylation (protein N-terminus) allowed as potential variable modifications. Peptide and protein probability was set at 90 and 99%, respectively and reported proteins required a minimum of two peptides.

## RESULTS

### FTO resides in both the nucleus and cytoplasm

**Figure 1:**
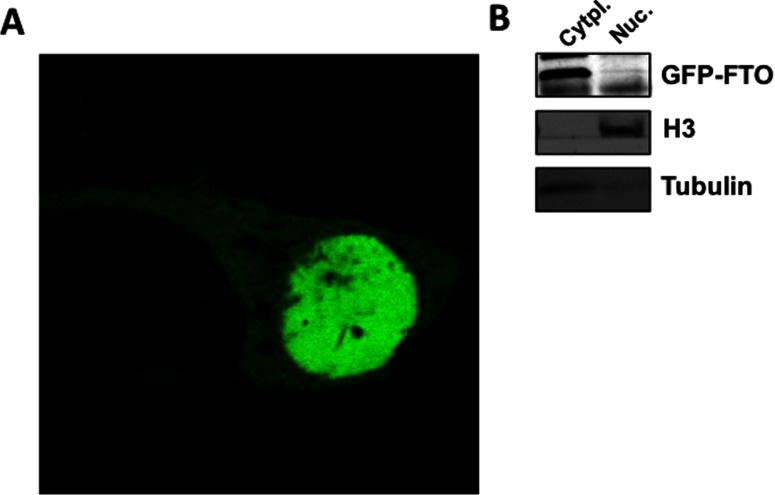
FTO is localized both in the nucleus and the cytoplasm (**A**) Representative confocal micrographs of live COS7 cells transiently transfected with GFP-FTO. (**B**) Cell extracts derived from GFP-FTO transfected COS7 cells were used to isolate nuclear and cytoplasmic fractions using the compartmental protein extraction kit from Millipore as per the manufacturer protocol.

To mitigate against the possibility of cell fixation disrupting the dynamic physiological balance of FTO's cellular compartmentalization, we performed live cell imaging of COS7 cells transiently expressing GFP-tagged FTO (GFP-FTO). We used confocal microscopy for the visualization of GFP-FTO expressed in live COS7 cells and found that GFP-FTO is present both in the nucleus and cytoplasm of live COS7 cells (Figure 1A). We then verified, using sub-cellular fractionation, that GFP-FTO expressed in COS7 cells is present both in the nuclear and cytoplasmic fractions (Figure 1B), as we had previously observed in MEFs and hypothalamic N46 cells [12].

### FTO shuttles between the nucleus and cytoplasm

Next, we asked whether FTO, once transported to a particular compartment, remains in place for the duration of its existence, or might it shuttle between the nucleus and cytoplasm? In order to address this we used the ‘fluorescence loss in photobleaching’ or FLIP technique [15]. This technique involves photo bleaching the fluorescence of a tagged protein (in this case GFP tagged to FTO) in a defined area of a live cell compartment, followed by measuring the fluorescent intensity in a second compartment of the same cell. A drop in fluorescence intensity in this second region would indicate a movement of the tagged protein from one compartment to the other [15].

**Figure 2:**
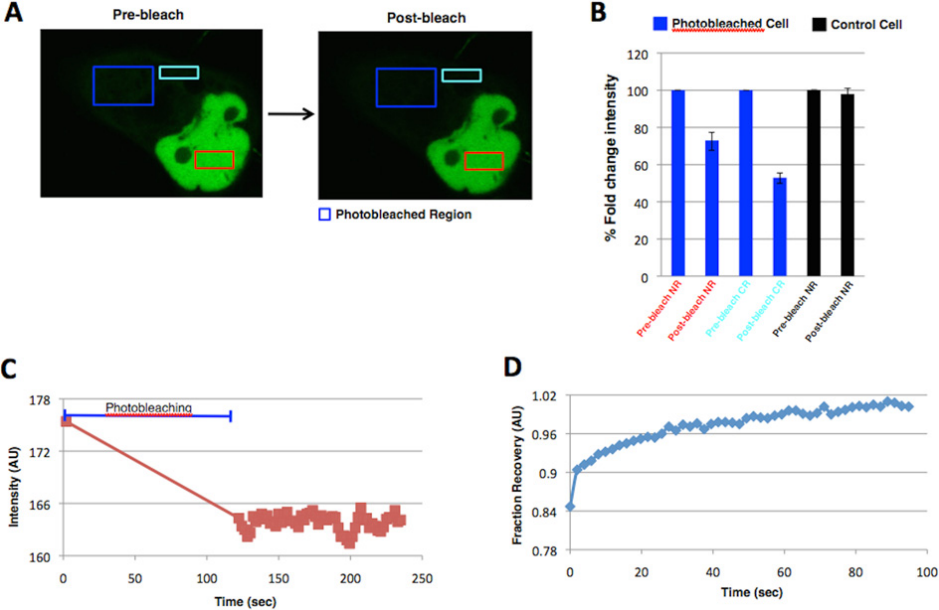
FTO shuttles from nucleus to the cytoplasm Representative confocal micrographs of GFP-FTO-transfected COS7 cells, before and after photobleaching, in the area outside (**A**) (blue boxes). Presented images are the first (before) and the last (after) in the time series. (**B**) Quantitation of GFP intensity obtained from different cells was performed and plotted as shown in the figure. All data are expressed as mean±S.E.M. NR, nuclear region; CR, cytoplasmicregion. (**C**) Fluorescence intensity in the photobleached region plotted as a function; (**D**) Normalized fluorescence intensity recovery (t1 half-13.5 s) after photobleaching in the photobleached region

Using this technique, we first performed cytoplasmic FLIP in live COS7 cells transiently transfected with GFP-FTO and noticed a decrease of fluorescence signal in the nucleus of the same cell that was subjected to cytoplasmic photobleaching (Figure 2A). We quantified the changes in the fluorescence intensity and found that after 2 min of photo bleaching in a cytoplasmic region (marked with dark blue box), there was almost a 30% decrease in the signal intensity of GFP-FTO in the nucleus (marked with red box) (Figure 2B) as well as 50% reduction of GFP intensity in the cytoplasmic region (marked with light blue box) adjacent to the photobleached region (Figure 2B). Post-photobleaching, we observed dynamic changes in the photobleached compartment (Figure 2C) as a result of GFP-FTO diffusion from the bleached area. There was no change in the GFP-FTO fluorescence intensity within the unbleached control cell (Figure 2B). The rate of fluorescence recovery in the photobleached region indicated a relatively high mobility of the protein in the cytoplasm (Figure 2D). This set of data indicates that GFP-FTO shuttles from cytoplasm to the nucleus.

**Figure 3:**
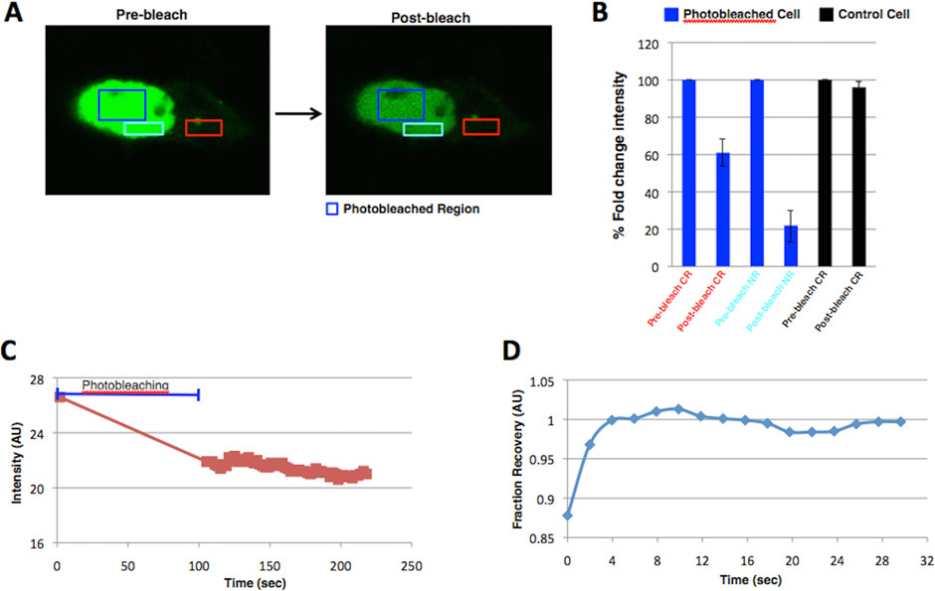
FTO shuttles from cytoplasm to the nucleus Representative confocal micrographs of GFP-FTO-transfected COS7 cells, before and after photobleaching, in the area inside the nucleus (**A**) (blue boxes). Presented images are the first (before) and the last (after) in the time series. (**B**) Quantitation of GFP intensity obtained from different cells was performed and plotted as shown in the figure. All data are expressed as mean±S.E.M. NR, nuclear region; CR, cytoplasmic region. (**C**) Fluorescence intensity in the photobleached region plotted as a function, (**D**) Normalized fluorescence intensity recovery (t1 half-1.4 s) after photobleaching in the photobleached region

In order to establish if GFP-FTO could also shuttle in a reciprocal fashion, we carried out a nuclear photobleaching experiment, while measuring the change in intensity of GFP-FTO in a cytoplasmic region (Figure 3A). We observed, as in the case of cytoplasmic FLIP, that nuclear photobleaching (marked with dark blue box) leads to a corresponding 40% decrease in the intensity of cytoplasmic GFP-FTO signal (marked with red box) (Figure 3B), and also an almost 80% drop of GFP intensity in the nuclear region (Figure 3B) adjacent to the photobleached region (marked with light blue box), indicating a high mobility of nuclear GFP-FTO (Figure 3C). This mobility of GFP-FTO is also apparent from fluorescence recovery after the photobleaching of the nuclear region (Figure 3D). We observed no effect on the GFP-FTO fluorescent intensity of the unbleached control cell (Figure 3B). This set of results confirms that in live cells, FTO is present in both the nucleus and cytoplasm, and points to a mobile fraction that shuttles between the two cellular compartments.

### XPO2 is a binding partner for FTO

Next we set out to investigate the possible mechanism underlying FTO shuttling within the cell. We performed a proteomic study, coupling IP of Flag-epitope tagged FTO from the lysates of transiently transfected HEK-293 cells with MS analysis, in order to identify potential FTO-binding protein partners, which could be involved in protein shuttling. In the MS analysis, we identified chromosome segregation 1-like, otherwise known as Exportin 2 (XPO2), in the Flag-FTO ‘pull-down’ sample, which was not present in the control vector transfected samples (Table 1). Exportins, together with importins, are a family of proteins that mediate the shuttling of proteins between the nucleus and cytoplasm and vice versa [16]. This observation was then validated using Western blots on Flag-FTO pull-down samples, where we could see co-IP (co-immunoprecipitation) of XPO2 with Flag-FTO (Figure 3A).

**Table 1:** List of FLAG-FTO-interacting proteins from MS analysis

Identified protein	Accession	Assigned spectra	Unique peptides
Exportin-2	XPO2_HUMAN	3	3
Methionyl-tRNA sythatase	SYMC_HUMAN	3	3
Programmed cell death 6-interacting protein	PDC6I_HUMAN	2	2
T-complex protein 1 subunit theta	TCPQ_HUMAN	4	4
T-complex protein subunit delta	TCPD_HUMAN	3	3
T-complex protein subunit eta	TCPH_HUMAN	3	3
Coatomer subunit delta	COPD_HUMAN	3	3
T-complex protein 1 subunit zeta	TCPZ_HUMAN	2	2
T-complex protein 1 subunit gamma	TCPG_HUMAN	2	2
Tyrosyl-tRNA synthetase	SYYC_HUMAN	2	2
Phosphoglycerate kinase 1	PGK_HUMAN	2	2

We further tested if endogenous FTO could interact with XPO2. For this purpose, we performed IP experiments of endogenous FTO from WT (wild-type) MEFs, and found that antibodies against FTO could indeed co-IP XPO2 (Figure 3B). These results confirm that XPO2 is an interacting protein partner of FTO. XPO2, under physiological conditions, has been reported to be present in both the nucleus and cytoplasm [14]. So we set out to confirm if FTO and XPO2 are both present in the same cellular compartment. For this we performed subcellular fractionation on MEF cells and found that like FTO, XPO2 is present in both the nuclear and cytoplasmic fractions (Figure 3C). The observation that FTO and XPO2 could be part of the same protein complex was further confirmed by isolation of native protein complexes using glycerol gradient fractionation. We prepared and loaded seven fractions, from low to high densities, on to an SDS–PAGE. We find that although XPO2 presence shows a peak in fractions six and seven, there are considerable amounts of XPO2 that are also present in the lower density fractions of one to four, along with FTO (Figure 3D). Altogether these results indicate that XPO2 interacts with FTO, and is a plausible candidate to be mediating the shuttling of FTO between nucleus and cytoplasm.

### N-terminal deleted FTO has altered cellular localization

**Figure 4:**
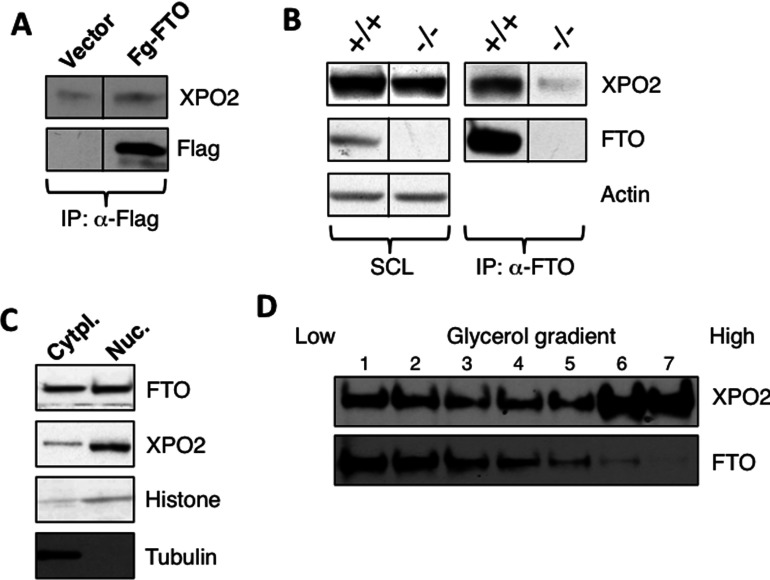
FTO interacts and co-localizes with XPO2 (**A**) HEK-293 cells transiently transfected with vector alone or Flag-FTO were cultured for 48 h. After 48 h, cells were harvested and lysed. Cell extracts obtained upon cell lysis were used for Flag IP using antibody against Flag protein as described in the methods. After IPs, the resulting immunoprecipitates were analysed by Western blotting using indicated antibodies. (**B**) Fto^+/+^ and Fto^−/−^ MEFs were grown for 24 h, after which cells were harvested and the resulting cell extracts were used for FTO IPs using the anti-FTO antibody as described in the Methods section. FTO immunoprecipitates were analysed by Western blotting. (**C**) Extracts derived from Fto^+/+^ MEFs were subjected to subcellular fractionation as in Figure 1(B). (**D**) Cell extracts were loaded onto the 10–45% (v/v) glycerol gradients as described in the methods. Different fractions collected were subjected to Western blotting.

X-ray crystallography of FTO has revealed a protein composed of two domains: an N-terminal domain carrying a catalytic core and a C-terminal domain of unknown structural homology [17]. In order to determine which of these domains might interact with XPO2, we generated N- and C-terminal deletion mutants of FTO. First we tested ability of these truncated versions of FTO to interact with XPO2 in Flag pull down experiments. We show that the C-terminal deleted mutant retains the ability to interact with XPO2 (Figure 4A). We were not, however, able to isolate the N-terminal deleted mutant, so are unable to comment on its ability to interact with XPO2 (Figure 4A). Next we examined the consequences of the N and C-terminal deletions, as well as the catalytically dead point R316Q mutation [8] on the cellular localization of FTO. To do this, we reverted back to live cell imaging in COS7 cells, where we observed that the C-terminal deletion mutant of FTO is expressed and retains the cell localization signature of WT-FTO (Figures 4B and 4C). The truncated GFP-FTO lacking its N-terminus however, appears to have a dysregulated cellular localization as compared with WT-FTO, losing its dominant nuclear staining (Figure 4D). Finally, we ask if the ability of FTO to shuttle between cellular compartments is reliant upon its catalytic ability as a demethylase? To analyse this we performed live cell imaging of COS7 cells expressing the previously reported catalytically inactive R316Q GFP-FTO. We observed that lack of demethylase activity does not affect the cellular localization of GFP-FTO (Figure 4E).

**Figure 5:**
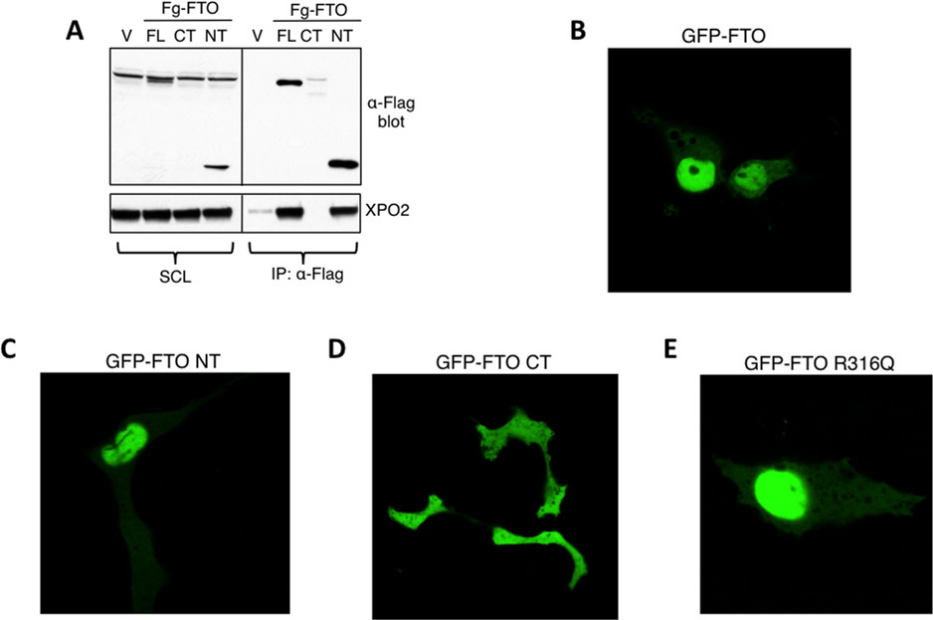
N-terminus of FTO through its interaction with XPO2 regulates FTO's cellular localization (**A**) HEK-293 cells over expressing vector alone or WT full-length (FL) FTO or truncated FTO C-terminal (CT) or truncated FTO N-terminal (NT) were cultured for 48 h. After 48 h cells were harvested and resulting cell extracts were subjected to Flag IP using antibody against Flag protein. Flag immunoprecipitates were analysed by Western blotting using indicated antibodies. *Non-specific protein band recognized by anti-Flag Ab in cell lysates. Representative confocal micrographs of COS7 cells transiently transfected with FL (**B**), NT (**C**), CT (**D**) and R316Q mutant (**E**) versions of FTO.

## DISCUSSION

We have previously described a critical role for FTO in the sensing of AAs and the regulation of growth and translation, which it does, in part, by interacting with amino-acyl tRNA synthetases [12]. However, as these synthetases are found primarily in the cytoplasm, this was difficult to reconcile with our initial observations, using immunofluorescence in fixed cells, that FTO was localized to the nucleus. Subsequently, in subcellular cell fractionation studies, we did see cytoplasmic localization of FTO by Western blot. Here, using live cell imaging, we show conclusively that FTO is indeed localized in both the nucleus and cytoplasm, and that there is a mobile fraction that shuttles between both compartments. These characteristics of FTO are more consistent with its role as a nutrient sensor and it would be interesting to explore if nutrients like AAs play any role in the regulation of FTO's cellular localization.

The enzymatic function of FTO is that of a nucleic acid demethylase with N6 methyl-adenosine [10] in mRNA and 3-methyl uracil [9,11] in ribosomal RNA as its prime substrates, with this ability to demethylate being critical for FTO's ability to sense AAs [12]. mRNA is transcribed and spliced in the nucleus, before being transported out to the endoplasmic reticulum to be translated, and it is currently unclear where and at which point FTO performs its demethylation of N6 methyl-adenosine. In fact, given what we show here, perhaps FTO plays some role in transporting a repertoire of mRNAs out of the nucleus. Ribosomal RNA, on the other hand, remains in the nucleus after transcription, where they are eventually combined together with ribosomal proteins that have been transported into the nucleus, to form ribosomes, making it likely that the demethylation of 3-methyl uracil occurs within the nucleus. This interaction between FTO and XPO2 could be a key role in the regulation of FTO function as a demethylase in the cell. In addition, FTO also acts as a demethylase on N6 methyl-adenosine, which mainly occurs in the mRNA population; thus the ability of XPO2 to shuttle FTO in and out of cytoplasm might play an important limiting factor in regulating the availability of FTO in a particular cell compartment to influence the extent of N6 methyl-adenosine demethylation on FTO mRNA substrates, which in turn would affect the rate of translation among those mRNA species.

FTO's ability to interact with XPO2, also known as chromosome segregation 1-like or CSE1L, tantalizingly provides a possible mechanism for how this shuttling of FTO in the cell might be happening. Proteins destined for transport to the nucleus typically carry an NLS (nuclear localization signal) that is recognized by the importin-α/β heterodimer, and is then translocated through the nuclear pore complex [16]. XPO2 was first identified as a necessary component to shuttle importin-α back to the cytoplasm, where it mediates another round of importin-α shuttling [18]. Subsequently, XPO2 was shown to also shuttle other molecules out of the nucleus [19]. Our data now include FTO as a possible candidate. We show that it is the N-terminus of FTO that binds to XPO2, and that the N-terminus of FTO in isolation retains the cellular localization of full-length FTO. The C-terminal portion of FTO has been trickier to manipulate and we have been unable to detect this deletion mutant using Western blot. We were however, able to visualize it using immunofluorescence and show that the cellular localization of C-terminal FTO is dysregulated as compared with the typical dominant nuclear staining in cells expressing WT-FTO.

There is then the question of the underlying mechanism linking the SNPs in the first intron of FTO to increased bodyweight. Smemo and colleagues report that the region encompassing the SNPs form long-range connections with the promoter of the downstream IRX3, and that the obesity-associated SNPs in *FTO* intron 1 are associated with expression of *IRX3*, but not *FTO*, in samples of human cerebellum [4]. They also show that *Irx3*-deficient mice have reduced body-weight, and thus argue that *IRX3* is a prime candidate to be the causative gene that links the risk SNPs to obesity [4]. We know, however, that FTO has an unequivocal role in the regulation of body-size and composition [6,7]. In addition, Stratigopoulos et al. have reported that mice heterozygous for targeted deletion in *Rpgrip1l*, which lies upstream of *FTO*, are fatter than WT controls, and that leptin signalling is diminished in the hypothalami of *Rpgrip1l*^+/−^ mice [5]. So it appears increasingly likely that the whole region plays an important role in determining body composition and weight.

In conclusion, we report here that FTO is present in both the nucleus and cytoplasm, and that there is a mobile fraction that shuttles between both cellular compartments, possibly through its interaction with XPO2. Further work will be required to delineate the relationship between IRX3, FTO and RPGRIP1L, and determine if they act in concert with each other.
